# Cost of Caregivers for Treating Hospitalized Diarrheal Patients in Bangladesh

**DOI:** 10.3390/tropicalmed4010005

**Published:** 2018-12-26

**Authors:** Abdur Razzaque Sarker, Marufa Sultana, Nausad Ali, Raisul Akram, Khorshed Alam, Jahangir A.M. Khan, Alec Morton

**Affiliations:** 1 Health Economics and Financing Research, International Centre for Diarrhoeal Disease Research, Bangladesh (icddr,b), 68, Shaheed Tajuddin Sarani, Dhaka 1212, Bangladesh; nausad.ali@icddrb.org (N.A.); raisul.akram@icddrb.org (R.A.); 2 Department of Management Science, University of Strathclyde, Glasgow G4 0QU, UK; alec.morton@strath.ac.uk; 3 School of Health and Social Development, Deakin University, Melbourne, Burwood VIC 3125, Australia; marufa@icddrb.org; 4 Nutrition and Clinical Services Division, International Centre for Diarrhoeal Disease Research, Bangladesh, Dhaka 1212, Bangladesh; 5 School of Commerce, and Centre for Health, Informatics and Economic Research, University of Southern Queensland, Toowoomba QLD 4350, Australia; Khorshed.Alam@usq.edu.au; 6 Department of Clinical Sciences, Liverpool School of Tropical Medicine, Liverpool L3 5QA, UK; Jahangir.Khan@lstmed.ac.uk

**Keywords:** caregiver, diarrheal disease, indirect cost, out of pocket payment, Bangladesh

## Abstract

Introduction: Diarrheal diseases are a global public health problem and one of the leading causes of mortality, morbidity and economic loss. The objective of the study is to estimate the economic cost of caregivers and cost distribution per diarrheal episodes in Bangladesh. Methods: This was a cross-sectional hospital-based study conducted in public hospitals in Bangladesh. A total of 801 diarrheal patients were randomly selected and interviewed during January to December 2015. Simple descriptive statistics including frequencies, percentage, mean with 95% CI and median are presented. Results: The overall average cost of caregivers was BDT 2243 (US$ 28.58) while only BDT 259 (US$ 3.29) was spent as out of pocket payments. Caregivers mostly spent money (US$ 1.63) for food, lodging, utility bills, and other lump sum costs followed by the transportation costs (US$ 1.57). The caregivers spent more (US$ 44.45) when they accompanied the patients who were admitted in inpatients care and almost 3.6 times higher than for out-patients care (US$ 12.42). Conclusions: The study delivers an empirical evidence to the health-care programmers and policy makers about the economic cost of caregivers during diarrheal treatment care, which should be accounted for in designing future diarrheal prevention programme.

## 1. Introduction

Diarrheal diseases remain one of the major causes of mortality and morbidity worldwide. Every year, approximately 2.39 billion diarrheal cases occurs globally, and in 2015 an estimated 0.53 million of under five children died due to diarrhoea [1,2] which was about nine per cent of all deaths among children under five years of age [3]. Specifically, incidence and case-fatality ratios are much higher in lower- and middle-income countries than developed economies [4]. Like many other developing countries, diarrhoea is an overwhelming public health problem in Bangladesh. Though the mortality due to diarrheal illness has significantly declined over the last decade, the morbidity remains stable in Bangladesh. In the last five years, approximately 12.9 million patients had visited health facilities for seeking care while at least 115 patients died [5]. However, numerous diarrheal cases occurred in community level and many of them were managed at household level and remain unreported, thus, the real diarrheal burden is still unknown.

Diarrhoea is one of the highly prevalent communicable diseases in Bangladesh. The diarrheal infection is common in all age groups, and children under five years of age suffer significantly in Bangladesh [6]. Bangladesh is located within a broad delta formed by the Ganges and Brahmaputra rivers of South Asia; the country is exceedingly flat, with low-lying land, subject to annual floods, and a natural disaster-prone area. Bangladesh is now undergoing a rapid urbanisation process. However, about one-third of the urban population live in urban slums, which are often considered high-risk areas for diarrheal infections [7]. Further, the diseases are highly sensitive to climate, showing seasonal variations in many places of the country [8]. Relative humidity and temperature are the other important factors that influence the rate of replication of different infectious organisms (e.g., bacteria and protozoa) and the survival of enteroviruses in the environment which cause diarrheal infections in Bangladesh [9]. diarrhoea is often referred as an alteration in normal bowel movement characterized by an increase in water content, volume, or frequency of stools [10]. A diarrheal episode is considered as the passage of three or more loose or liquid stools in 24 h prior to presentation for care, which is considered the most practicable definition among children and adults [11]. If the disease lasts “more than 7 days” and “at least 14 days” then the terms “prolonged” and “persistent” diarrhoea are used respectively [12,13]. diarrhoea is caused by many infectious organisms, including bacteria (e.g., *Escherichia coli*, *Vibrio chollerae Shigella*, *Salmonella*), viruses (e.g., *Rotavirus*, *Adenovirus*, *Norovirus*) and parasites (e.g., *Entamoeba Histolytia*, *Giardia Lamblia*). Poor sanitation systems, lack of potable water and inadequate personal hygiene are important risk factors for diarrheal disease which is accountable for up to 90% of all diarrheal cases [14], although it is the most prevalent disease that affects all irrespective of socio-economic status. The disease can be prevented and managed at household level with low cost oral rehydration therapy (ORT), however, the patient frequently visits the health facilities which imposes a substantial economic burden for the affected households [15,16]. Recent study showed that by controlling diarrheal diseases households could save approximately US$ 136.03 million in Bangladesh which usually exhausted for receiving treatment [17].

Diarrheal diseases are one of the major causes of hospitalization among under-five children in Bangladesh. According to the latest hospital-based surveillance in Bangladesh, childhood diarrheal diseases were responsible for 40% and 18% of hospital admissions in sub-district and district level hospitals respectively, while at least 7% of under-five children were admitted to the medical college hospitals [5]. Although diarrheal diseases can be managed by low-cost interventions, however, an earlier study observed that about 75% of the under five children receive treatments from formal healthcare providers [18]. However, the treatment seeking pattern for diarrheal disease varies to a greater extent for children than for adults as children frequently visit the local private practitioners before hospitalization which eventually is reflected in its treatment costs [18,19].

There are numerous studies focusing on the economics of diarrheal disease around the world however, research focusing on the caregivers’ cost during the diarrheal episode is still limited globally [20,21,22,23,24,25]. A hospital-based study captured the unit cost of inpatients and outpatients from providers’ points of view rather than household perspectives in Asia [26]. Another hospital-based study also reported the cost for treating <5 diarrheal patients in Bangladesh but failed to capture all component (e.g., laboratory cost and income loss) of costs [27]. None of the studies specifically focused on the caregivers’ point of views. Caregivers’ time and direct expenditure are important cost component and have an impact on the livelihoods of families and particularly for those who live further away from treatment facilities [17,28,29,30]. To the best of our knowledge, this is the first study to analyse the distribution of caregivers’ cost in Bangladesh, although caregivers’ time and financial resources could upturn the overall cost of treatment for household perspective. Earlier hospital-based survey indicated that approximately 44 percent of the total diarrheal patients required hospitalization and the remaining patients received outpatient services [17]. However, in both cases, the caregiver’s involvement is a common phenomenon. In this aspect, the objective of the study is to estimate the average cost of caregivers and cost distribution during diarrheal episodes in Bangladesh. Given the current focus on the treatment cost from household perspective, the study aims to analyse both the costs of outpatients and inpatients during diarrheal infections. We expect that our findings will help policy makers to evaluate and design the diarrhoea related preventive or promotive health intervention at the household level.

## 2. Materials and Methods

### 2.1. Design and Study Population

This was a cross-sectional descriptive study conducted in public hospitals in Bangladesh. Public hospitals play a major role in providing treatment to the relatively large population with low cost, as those hospitals are highly subsidized and financed by the government of Bangladesh. Thus, a large number of patients irrespective of socio-economic strata frequently seek care from public hospitals. A total of 801 diarrheal patients were randomly selected and interviewed during January to December 2015. Respondents of this study were adult caregivers or economic contributors or adult patients. The study was conducted from a caregiver’s perspective which means all types of caregiver’s costs were identified, measured and valued [31].

### 2.2. Cost Estimates

The aim of this study was to analyse the caregivers’ cost associated with the diarrheal treatment. In the study caregivers can be mother, father, spouses, descendants, siblings or close relative, who have no training in child health or education except their own experience and accompany during the hospitalization and aged 18 and above [19]. To estimate the cost of caregivers, both direct and indirect costs were captured. *Out of pocket costs* were defined as expenditure by caregivers for themselves which includes transportation, lodging, food items, informal payment, utility bills and other associated payments which was not directly linked with the treatment of patients. *Indirect cost* was considered as the income losses, as well as productivity losses of caregivers because of travel and stay at the health centre and costs due to absence from work. Self-reported wage rates were used for estimating the income losses. The inclusion of caregiving time based on the assumption that time dedicated to caregiving may represent foregone non-market activities such as education, household chores, child care, and leisure or domestic work [32,33]. Productivity costs were estimated using the human capital approach [34]. To capture the productivity losses for non-market activities, the minimum wage rate of Bangladesh according to the national level was considered. Caring for a patient with diarrhoea may have in negative impact on the emotional and physical conditions of the caregivers which was not captured in the previous studies [33,35]. Furthermore, time cost of visitors and extra irregular expense borne by the caregivers and visitors were not included in the analysis.

### 2.3. Data Collection

Data were collected by face-to-face interviews during discharge from the hospitals. Respondents were adult patients or the accompanying persons who were most familiar with the costs incurred during the treatment of the patient. A research assistant reviewed patient’s records, and data extraction forms were updated daily until the discharge of the patients. A telephone interview was also conducted for taking necessary information within one week after discharge from the hospital. Questions were asked regarding transportation, expenses during the hospitalization, and losses of wages resulting from absence from work.

### 2.4. Data Analysis

Data analysis was performed using Microsoft Excel and Stata/SE 13.0 (StataCorp, College Station, TX, USA). Simple descriptive statistics including frequencies, percentage, mean (95% CI) and median were presented in local currency, i.e., Bangladeshi Taka (BDT) and US dollars (US$) applying the exchange rate (US$ 1 = 78.5 BDT) during the year of the survey; mid-2014–mid-2015 [36].

### 2.5. Ethical Approval

The research protocol of this study, PR 13064 was approved by the Institutional Review Board of the International Centre for Diarrheal Disease Research, Bangladesh (icddr,b). Informed consent was obtained from all respondents before data collection.

## 3. Results

### 3.1. Background Characteristics of Study Participants

The background characteristics of patients and their caregivers are presented in Table 1. A higher proportion of the diarrheal patients were children aged less than five years (57%) followed by adult patients aged 20 to 64 years (25%). The proportion of male and female patients were almost same (50%), and utilization of inpatient care service was slightly higher (52%) than outpatient services. Except the children aged less than five years (57%), housewives (16%), and students (10%) were two most vulnerable occupational groups for diarrheal infection. Parents (64%) were the most common person caring for their children during diarrheal treatment, indeed most of the caregivers were housewives (90%). The monthly income and expenditure of the households were BDT 19,603 (US$ 250) and BDT 15,470 (US$ 197) respectively, while the average last three months healthcare expenditure was BDT 5191 (US$ 66) considering last three months preceding to the survey.

### 3.2. Average Caregivers’ Cost

The average caregivers’ economic cost and its distribution were reported in Table 2. The overall average caregivers’ cost for attending with their diarrheal patients was BDT 2, 243 (US$ 28.58) while only BDT 258.57 (US$ 3.29) was spent as out of pocket payment. Among this expenditure, caregivers spent most for food, lodging, utility bills, and other lump sum costs jointly (BDT 127.87 or US$ 1.63) followed by the transportation costs (BDT 123.29 or US$ 1.57). As with overall care, the cost distribution pattern was similar for inpatient care but different for outpatient care where caregivers spent more for transportation cost than food and lodging purpose (Table 2). Indeed, caregivers spent more when they accompanied the inpatients than the outpatients and this amount was almost 3.6 times higher than outpatients (BDT 3,490 or US$ 44.45 V/S BDT 975 or US$ 12.42).

### 3.3. Distribution of Caregivers’ Cost by Socio-Demographic Characteristics

Considering the age group of patients, the average total cost of caregivers ranged from BDT 2529 (US$ 32.22) to BDT 4407 (US$ 56.14) for inpatient care. For inpatient care, caregivers’ cost was significantly higher (BDT 4407 or US$ 56.14) when they accompanied under-five children followed by the older aged (64+) patients (BDT 3777 or US$ 48.11). A similar cost distributional pattern was observed regarding direct and indirect cost of caregivers for both the inpatient and outpatient care. Caregivers’ average total expenditure was slightly higher (BDT 3616 or US$ 46.06) for male patients than female patients (BDT 3357 or US$ 42.76), and a similar scenario was observed for direct and indirect cost for inpatient care (Table 3). However, this situation was quite different for outpatient care where cost of caregivers was comparatively higher for female out patients. In poorest quintile, the average total cost of caregivers for inpatients were BDT 3377 (US$ 43.02) while that for the richest quintile were BDT 3896 (US$ 49.62). The direct cost of caregivers for poorest quintile (BDT 379 or US$ 4.82) was lower than that of richest (5th) quintile (BDT 519 or US$ 6.61). A similar pattern was also observed for indirect costs (Table 3). However, such a pattern was not observed when they accompanied the patients for out-patient care.

### 3.4. Caregivers’ Time

**Figure 1:**
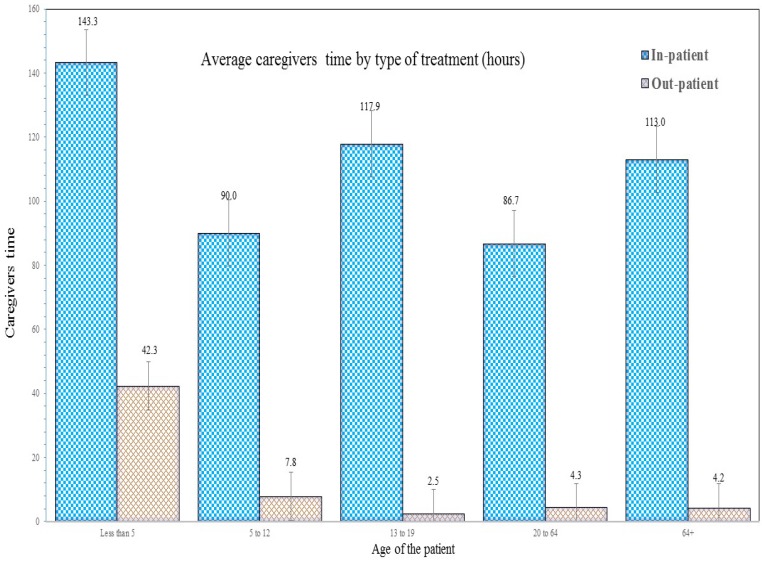
Average caregivers’ time by type of treatment (h).

The average time of caregivers during inpatient and outpatients care was 113 h and 7.8 h per episode respectively, while the caregiving time is higher when they accompanied inpatients (Figure 1). Furthermore, caregivers spent more time for caring under-five children as sometimes multiple caregivers attended during their course of treatment.

## 4. Discussion

Diarrheal diseases remain a crucial public health concern in Bangladesh as large number of people (approximately 2.7 million) utilize the healthcare facilities annually due to diarrheal infection, which leads to an excessive pressure on the country’s health system. Furthermore, households spend significant resources for the treatment purpose both directly and indirectly whereas caregivers’ expenditures and indirect cost are the important components of costs during the treatment courses. Caregivers’ support and services are vital for managing and treating patients particularly for under-five children and older aged and sometimes are especially essential when the patients felt difficulties to speak for him or herself [37]. Caregiving during diarrheal infections may help the patients for their daily assistance (e.g., dressing, toileting and child care), monitoring activities (e.g., financial transaction and administration of medication) and emotional support [38]. Caregivers’ help and support are often vital for inpatient cares which often associated with the caregivers’ burden both physically and economically [39]. In the present study we aimed to investigate the caregivers’ direct and indirect cost due to their attendance and supervision during the treatment of diarrheal patients in tertiary district public hospitals in Bangladesh.

Along with the health burden, diarrheal infections have potential economic impact on diarrhoea affected households. A number of studies focused on the economic burden of diarrheal infections in various countries [20,21,22,23,24,25], but the knowledge about caregivers costs of a full diarrheal episode are still limited in resource-poor settings, particularly in the context of Bangladesh. Various hospital-based costing studies were conducted in Bangladesh to estimate treatment cost per patients from the provider’s perspective [26,27]. However, those studies conducted either in a single public hospital or in a surveillance hospital. Further, all components of costs (e.g., income/productivity loss of patients, caregivers) were not included in their estimation. A multi-country analysis was conducted in ASEAN context focusing on childhood diarrhoea [40]. The study found that the mean household costs were US$ 1.82, US$ 6.47, and US$ 3.33 for Bangladesh, Pakistan, and India, respectively. However, the study excluded the treatment cost for adults and not explained the caregiver’s perspectives. Various slum-based diarrheal costing studies were also conducted in Bangladesh [41,42]. However, none of the above studies represented the country scenario or considered the cost burden from caregiver’s perspective. Our study demonstrated that the average total cost of caregivers was US$ 28.58 per diarrheal episode whereas the average total cost for caring inpatients and outpatient was US$ 44.45 and US$ 12.42, respectively. Our estimation suggested that annual caregivers’ cost during diarrheal infections to be US$ 74.71 million which constituted for 5.34% of the total health expenditure in Bangladesh [43]. The out of pocket cost incurred by the caregivers was mainly for purchasing foods and lodging purposes while a vast indirect cost was also incurred due to their absence from daily productive activities. An economic analysis regarding cost of caregivers of DAZT (diarrhoea alleviation through zinc and oral rehydration salts therapy) program was conducted in Gujrat, India and found that the average economic cost could be incurred up to US$ 4.04 per episode for rural under-five children [16]. Several studies also indicated that caregivers’ cost is important aspect for households which might be increased the total treatment cost during diarrheal episode from societal perspective [17,33,44,45]. An earlier study in this context observed that the additional caregiver cost could increase the average treatment costs up to US$ 110.51 per episode of diarrheal infection [17]. In our study we have found that caregivers’ cost was highest when they provided care to the under-five children as they were the most vulnerable for diarrheal infections [18,46]. In another earlier study to this context, it was shown that healthcare seeking behaviour of households were quite different regarding their young members when exposed diarrheal infections and they were often taken to the private providers or clinics (e.g., private practitioners) before hospitalization which was not observed for adult patients [33]. Furthermore, household members particularly parents became terrified and worried when they found their young family members are at risk of ill-health which is reflected in their direct and indirect costs as both the time and in-person involvement required are generally comparatively more for children than adult patients [19].

Like previous studies [17,19,33,44,45] we also observed that households from the poorest quintile spent less than richest quintile because of their affordability but faced highest cost burden than richest quintile although the diarrheal prevalence are the highest among household members of the poorest quintile [17,18]. For such consequences, many diarrheal cases are inadequately managed at household level which eventually pushes them to the point where the patients’ life is endangered [47]. An earlier study observed that during diarrheal episode households often sought care from formal (e.g., qualified doctor, hospital) and informal providers (e.g., tradition healers, unqualified village doctors), even about 23% of diarrheal patients did not seek formal care, although rapid and proper treatment is essential to avoid excessive costs of households and adverse health events [16,18]. Although we did not particularly capture the source of financing for caregivers, an earlier study indicated that regular income and borrowing were the two common coping strategies for diarrheal treatment, which is crucial for Bangladesh as the social health protection scheme not yet implemented and out-of-pocket is the main payment strategy (67%) for seeking healthcare [17,43].

There are various proven interventions for preventing diarrheal infections. However, the vast number of diarrheal cases could be prevented by implementing water, sanitation, and hygiene (WASH) strategies [48,49]. A couple of systematic reviews confirmed that hand washing [50] and point-of-use water treatment [51] were effective interventions for reducing diarrhoeal diseases. Cairncross and colleagues identified that the risk of diarrhoea could be reduced by implementing hand washing with soap (48%), improved water quality (17%) and adequate disposal of human waste (36%) in many developing countries [52]. Since diarrhoea is manifested by dehydration in most of the cases, ORS has proven as effective treatment without any significant adverse effect [53]; and as much as 69% of diarrhoea-related mortality could be prevented by administrating ORS and recommended home fluids [54] which contribute to decrease length of stay or even hospitalization thus reduces the excessive cost during diarrheal treatment. The promotion of exclusive breastfeeding [55], and complementary feeding practices [56] might strengthen the immune system of children and reduce the prevalence of childhood diarrhoea. Although WASH programs, such as improvements in water/sanitation infrastructure and personal hygiene contribute to decline the transmission of enteric pathogens, vaccine can hasten the waning of diarrhoea-related mortalities and morbidities, particularly in epidemic and endemic settings [57]. It was reported that nearly one-third of the episodes of severe diarrhoea could be prevented by introducing vaccination against rotavirus and cholera infections [58]. Policy makers should consider the caregivers cost found in our study for justifying the future diarrheal prevention programmes.

The study has some limitations when interpreting these findings. There may be some recall bias as data were collected after receiving treatment although we have tried to capture all the information within the two weeks of discharge from hospital. The limitations to this study also include the design; as a cross-sectional, hospital-based study it is unable to capture the seasonal variation of caregivers cost as the incidence of diarrheal disease usually peaks during the hot and winter seasons in Bangladesh [59]. The cost of caregivers relies heavily on households’ treatment seeking patterns, resources and ability to pay which were not captured [60]. Further, we could not compare the treatments cost using a pairwise matching approach due to the non-availability of matching data. Similarly, the present study was also unable to observe the cost of adverse effects and emotional effects (such as anxiety and tiredness) of caregivers during the diarrheal effects. All these issues should be taken into account in future research.

## 5. Conclusions

Diarrhoea is still an overwhelming public health issue in Bangladesh. Our study observed that the average cost of caregivers for treating each episode of diarrhoea was US$ 28.58 (BDT 2,243). Regarding the types of services, the caregivers spend almost 3.6 times higher for inpatient care than outpatients and caregivers’ cost was significantly higher when they accompanied under-five children and older aged patients. The study delivers empirical evidence for the health-care programmers and policy makers about the economic cost of caregivers during diarrheal treatment care. However, the additional caregivers’ cost is likely to be even higher than what has been observed in this empirical study. These findings should be taken into account in designing future diarrheal prevention programmes in Bangladesh and other developing countries.

## Figures and Tables

**Table 1:** Distribution of socio-demographic characteristics of diarrheal patients (*N* = 801).

Variables	n or mean	% or SD	95% CI
Patient age in years (%)			
Less than 5	460	57.43	(53.97, 60.82)
5 to 12	52	6.49	(04.98, 08.43)
13 to 19	38	4.74	(03.47, 06.46)
20 to 64	199	24.84	(21.97, 27.96)
64+	52	6.49	(04.98, 08.43)
Gender (%)			
Male	404	50.44	(46.97, 53.90)
Female	397	49.56	(46.10, 53.03)
Types of patient (%)			
Inpatient	404	50.44	(46.97, 53.90)
Outpatient	397	49.56	(46.10, 53.03)
Patient education (%)			
Not applicable (Child < 5 years)	458	57.18	(53.72, 60.57)
No formal education	95	11.86	(09.79, 14.29)
Up to primary	112	13.98	(11.74, 16.57)
Secondary	103	12.86	(10.71, 15.37)
Higher	33	4.12	(02.94, 05.74)
Patient occupation (%)			
Not applicable (Child < 5 years)	458	57.18	(53.72, 60.57)
Housewife	130	16.23	(13.83, 18.95)
Students	83	10.36	(08.43, 12.68)
Self-employment	49	6.12	(04.65, 08.01)
Unemployed	7	0.87	(00.42, 01.82)
Salaried employee	31	3.87	(02.73, 05.45)
Business	18	2.25	(01.42, 03.54)
Others	25	3.12	(02.12, 04.58)
Mother education level (%)			
No formal education	270	33.71	(30.51, 37.06)
Up to primary	199	24.84	(21.97, 27.96)
Secondary	274	34.21	(30.99, 37.57)
Higher	58	7.24	(05.64, 09.26)
Father education level (%)			
No formal education	278	34.71	(31.48, 38.08)
Up to primary	191	23.85	(21.01, 26.93)
Secondary	241	30.09	(27.00, 33.36)
Higher	91	11.36	(09.34, 13.76)
Patient-respondent relationship (%)			
Parent	509	63.55	(60.14, 66.82)
Sibling	17	2.12	(01.32, 03.39)
Grandparent	17	2.12	(01.32, 03.39)
Relative	29	3.62	(02.53, 05.17)
Spouse	52	6.49	(04.98, 08.43)
Offspring	49	6.12	(04.65, 08.01)
Others (i.e., neighbour, friend, self)	128	15.98	(13.60, 18.69)
Caregivers occupation (considered multiple responses)			
Housewife	702	90.00	-
Students	73	09.36	-
Self-employment	114	14.62	-
Unemployed	31	03.97	-
Salaried employee	103	13.21	-
Business	118	15.13	-
Others	130	16.67	-
Household size (%)			
Less than 3	32	4.00	(02.84, 05.60)
3 to 5	458	57.18	(53.72, 60.57)
More than 5	311	38.83	(35.50, 42.26)
Household size (Mean, SD)	5.59	02.93	(05.59, 02.93)
Patient household monthly income in BDT (Mean, SD)	19,603	26,642	(17,756, 21,451)
Patient household monthly expenditure in BDT (Mean, SD)	15,470	10,702	(14,727, 16,212)
Overall healthcare expenditure last 3 months in BDT (Mean, SD)	5191	17,745	(3961, 6422)
Income quintile in BDT (Mean, SD)			
Poorest quintile	190	7042	(6786, 7298)
2nd	168	10,938	(10,789, 11,086)
3rd	123	14,638	(14,513, 14,763)
4th	178	20,288	(19,893, 20,683)
Upper quintile	142	50,106	(41,466, 58,746)

Note: SD: Standard Deviation; CI: Confidence Interval.

**Table 2:** Distribution of average caregivers’ cost of diarrheal treatment for tertiary level hospital (*N* = 801) BDT (US$).

Cost	Parameter	Overall Cost		Inpatient Cost		Outpatient Cost	
		Average	SD	Average	SD	Average	SD
Out-of-pocket cost	Transportation cost	123.29 (01.57)	213.88 (02.72)	189.18 (02.41)	242.4 (03.09)	56.24 (0.72)	153.89 (01.96)
	Informal payment (e.g., tips)	07.40 (0.09)	22.65 (0.29)	14.23 (0.18)	29.94 (0.38)	0.45 (0.01)	05.35 (0.07)
	Caregiver’s payment	0.01 (0.00)	0.35 (0.00)	0.00 (0.00)	0.00 (0.00)	0.03 (0.00)	0.50 (0.01)
	Caregivers expenditure (e.g., food, lodging)	127.87 (01.63)	456.8 (05.82)	228.99 (02.92)	438.94 (05.59)	24.97 (0.32)	452.15 (05.76)
Total direct cost		258.57 (03.29)	541.07 (06.89)	432.4 (05.51)	542.34 (06.91)	81.69 (01.04)	479.48 (06.11)
In-direct cost	Caregivers income loss	1985.21 (25.29)	2467.44 (31.43)	3057.31 (38.95)	2540.84 (32.37)	894.2 (11.39)	1834.02 (23.36)
Total cost		2243.38 (28.58)	2756.87 (35.12)	3489.67 (44.45)	2789.05 (35.53)	975.11 (12.42)	2059.31 (26.23)

Note: 1 US$ = 78.5 BDT; SD: Standard Deviation.

**Table 3:** Distribution of average care giver expenditure by socio-demographic characteristics (*N* = 801), BDT, US$.

Variables	In-Patients						Out-Patients					
	Total Cost		Direct		Indirect		Total Cost		Direct		Indirect	
	Average	SD	Average	SD	Average	SD	Average	SD	Average	SD	Average	SD
Patient age in years												
Less than 5	4407 (56.14)	2639 (33.62)	558 (7.11)	566 (7.21)	3848 (49.02)	2400 (30.57)	1173 (14.94)	2256 (28.74)	94 (1.20)	535 (6.82)	1079 (13.75)	2006 (25.55)
5 to 12	2529 (32.22)	1619 (20.62)	411 (5.24)	532 (6.78)	2119 (26.99)	1563 (19.91)	246 (3.13)	230 (2.93)	34 (0.43)	43 (0.55)	212 (2.70)	217 (2.76)
13 to 19	3405 (43.38)	2884 (36.74)	261 (3.32)	305 (3.89)	3144 (40.05)	2808 (35.77)	58 (0.74)	98 (1.25)	34 (0.43)	25 (0.32)	44 (0.56)	72 (0.92)
20 to 64	2714 (34.57)	2741 (34.92)	318 (4.05)	388 (4.94)	2396 (30.52)	2525 (32.17)	132 (1.68)	159 (2.03)	40 (0.51)	35 (0.45)	110 (1.40)	128 (1.63)
64+	3777 (48.11)	2833 (36.09)	568 (7.24)	852 (10.85)	3209 (40.88)	2441 (31.10)	130 (1.66)	183 (2.33)	40 (0.51)	18 (0.23)	110 (1.40)	155 (1.97)
*P-value*	<0.001		<0.001		<0.001		<0.001		0.89		<0.001	
Gender												
Male	3616 (46.06)	3081 (39.25)	437 (5.56)	616 (7.85)	3179 (40.50)	2774 (35.33)	953 (12.14)	1896 (24.15)	67 (0.85)	213 (2.71)	887 (11.29)	1816 (23.14)
Female	3357 (42.76)	2446 (31.16)	428 (5.45)	454 (5.78)	2929 (37.32)	2271 (28.92)	997 (12.7)	2213 (28.19)	96 (1.23)	642 (8.18)	902 (11.49)	1856 (23.64)
*P-value*	0.35		0.87		0.32		0.83		0.54		0.93	
Household size												
Less than 3	2360 (30.06)	2148 (27.36)	244 (3.11)	262 (3.34)	2116 (26.96)	2036 (25.93)	52 (0.67)	81 (1.03)	20 (0.25)	12 (0.15)	44 (0.56)	69 (0.88)
3 to 5	3372 (42.95)	2659 (33.88)	423 (5.39)	547 (6.97)	2949 (37.56)	2367 (30.16)	1096 (13.96)	2343 (29.84)	96 (1.22)	594 (7.57)	1000 (12.74)	2037 (25.95)
More than 5	3794 (48.33)	2974 (37.88)	471 (6.00)	563 (7.18)	3323 (42.33)	2763 (35.20)	790 (10.06)	1419 (18.07)	57 (0.73)	93 (1.18)	733 (9.33)	1406 (17.92)
*P-value*	0.03		0.13		0.05		0.20		0.71		0.20	
Income quintile												
Poorest quintile	3377 (43.02)	2620 (33.37)	379 (4.82)	522 (6.65)	2998 (38.19)	2363 (30.11)	1074 (13.68)	2649 (33.75)	125 (1.59)	883 (11.25)	950 (12.10)	2065 (26.30)
2nd	3289 (41.90)	2498 (31.82)	433 (5.51)	469 (5.98)	2856 (36.39)	2304 (29.35)	790 (10.06)	1424 (18.15)	51 (0.65)	70 (0.89)	740 (9.43)	1393 (17.75)
3rd	3568 (45.46)	2468 (31.44)	417 (5.31)	518 (6.60)	3151 (40.14)	2296 (29.25)	821 (10.46)	1718 (21.89)	48 (0.61)	87 (1.11)	774 (9.86)	1708 (21.75)
4th	3334 (42.47)	2880 (36.69)	413 (5.26)	473 (6.03)	2921 (37.21)	2599 (33.11)	1096 (13.96)	1975 (25.16)	77 (0.98)	285 (3.63)	1019 (12.99)	1875 (23.89)
Upper quintile	3896 (49.62)	3335 (42.48)	519 (6.61)	695 (8.86)	3377 (43.02)	3035 (38.66)	1033 (13.16)	2114 (26.93)	89 (1.14)	162 (2.06)	945 (12.03)	2052 (26.14)
*P-value*	0.62		0.54		0.70		0.81		0.83		0.84	
Overall	3490 (44.45)	2789 (35.53)	432 (5.51)	542 (6.91)	3057 (38.95)	2541 (32.37)	975 (12.42)	2059 (26.23)	82 (1.04)	479 (6.11)	894 (11.39)	1834 (23.36)

Note: 1 US$ = 78.5 BDT; SD: Standard Deviation.
